# Relation of Health-Related Quality of Life with Glycemic Control and Use of Diabetes Technology in Children and Adolescents with Type 1 Diabetes: Results from a National Population Based Study

**DOI:** 10.1155/2022/8401328

**Published:** 2022-11-03

**Authors:** Heiko Bratke, Eva Biringer, Hanna D. Margeirsdottir, Pål R. Njølstad, Torild Skrivarhaug

**Affiliations:** ^1^Department of Pediatrics, Haugesund Hospital, Fonna Health Trust, Haugesund, Norway; ^2^Center for Diabetes Research, Department of Clinical Science, University of Bergen, Bergen, Norway; ^3^Oslo Diabetes Research Centre, Oslo, Norway; ^4^Department of Research and Innovation, Fonna Health Trust, Haugesund, Norway; ^5^Division of Childhood and Adolescent Medicine, Oslo University Hospital, Oslo, Norway; ^6^Child and Youth Clinic, Haukeland University Hospital, Bergen, Norway; ^7^University of Oslo, Institute of Clinical Medicine, Faculty of Medicine, Oslo, Norway; ^8^The Norwegian Childhood Diabetes Registry, Division of Childhood and Adolescent Medicine, Oslo University Hospital, Oslo, Norway

## Abstract

**Objective:**

The primary aim was to analyse the association between diabetes-specific health-related quality of life (HRQOL) and HbA1c in children and adolescents with type 1 diabetes. The secondary aims were to evaluate the associations between diabetes-specific HRQOL and age, sex, diabetes duration, and the use of diabetes technology in diabetes treatment. *Research Design and Methods*. Children with type 1 diabetes (10-17 years, *N* = 1,019) and parents (children <10 years, *N* = 371; 10-17 years, *N* = 1,070) completed the DISABKIDS diabetes-specific questionnaire (DDM-10) as part of the 2017 data collection for the Norwegian Childhood Diabetes Registry. The DDM-10 consists of two subscales—‘impact' and ‘treatment'—with six and four items, respectively. In the linear regression models, the items and subscales were outcome variables, while HbA1c, age, sex, diabetes duration, insulin pump use, and continuous glucose monitoring (CGM) system use were predictor variables.

**Results:**

Lower HbA1c measurements and male sex were associated with higher HRQOL scores on both DDM-10 scales in the age group 10-17 years, but not in children under 10 years. Parents gave lower HRQOL scores than children in the 10-17 age group. Insulin pump and CGM use were not significantly associated with HRQOL on the impact and treatment scale.

**Conclusions:**

Low HbA1c and male sex are significantly associated with high HRQOL in children aged 10-17 with type 1 diabetes, but the use of diabetes technology is not positively associated with HRQOL. Differences in child- and parent-reported scores imply that parents might both over- and underestimate their child's HRQOL.

## 1. Introduction

Previous studies have shown that optimal glycemic control, as reflected by a low glycosylated haemoglobin A1c (HbA1c), is associated with a better health-related quality of life (HRQOL) [1, 2]. Research has also evaluated whether the use of continuous subcutaneous insulin infusion (CSII) [3–8] and the use of continuous glucose monitoring (CGM) [9] are associated with higher quality of life (QoL), but such studies on the associations between QoL and HbA1c or diabetes technology could have limitations such as small or heterogeneous study samples or short follow-up time. A large percentage of children and adolescents in these studies did not reach the HbA1c goal of the International Society for Pediatric and Adolescent Diabetes. Furthermore, earlier CSII and CGM systems were less user-friendly and less sophisticated, and their usage was not as common as today and was often reserved for special indications. A meta-analysis by Rosner and Roman-Urrestarazu based on earlier studies from 2003-2018 concluded that the use of CSII was not associated with significant changes in QoL over time [6].

Over the last decade, CSII and CGM usage has increased steeply in many developed countries [3, 10, 11]. Norway was an early adaptor of diabetes technology, with a steady increase in the use of CSII and CGM, leading to 74% of the pediatric age group using CSII and 52% using CGM in 2017 [12]. Recently, national and international childhood diabetes registries have reported improved metabolic control in type 1 diabetes in terms of lower mean HbA1c and a higher percentage of participants reaching recommended HbA1c values [13–15]. The Norwegian Childhood Diabetes Registry (NCDR) captures nearly all youth with type 1 diabetes, with a completeness of 98% [12]. Froisland et al. [16] reported for the first time the HRQOL among Norwegian children and adolescents with type 1 diabetes using the newly validated DISABKIDS module. In their study based on data from 2010-2011, lower HbA1c was associated with higher HRQOL. Furthermore, they reported that the use of insulin pumps was not associated with a higher HRQOL [17].

Norway has one of the world's highest incidences of type 1 diabetes with 37.1 per 100.000 person-years in the age group 0-14 years [12]. From 2010 to 2017, the national mean HbA1c of children 0-18 years registered in the NCDR decreased from 8.6% (70 mmol/mol) to 7.9% (62 mmol/mol) [12]. At the time of the study, the actual ISPAD HbA1c target was 7.5% (58 mmol/mol) [18]. The percentage of individuals reaching HbA1c at target has increased from 18% in 2010 to 39% in 2017. Simultaneously, the incidence of hospitalizations due to diabetic ketoacidosis (DKA) decreased from 4% to 3% of children in the NCDR, and the incidence of severe hypoglycemia from 6% to 3% (unpublished NCDR data). However, the association between HbA1c and HRQOL has not been investigated after these improvements in diabetes care occurred. Studies on HRQOL in nation-based pediatric diabetes populations, using modern insulin delivery and glucose monitoring devices, are lacking. Central questions to ask are the following: has HRQOL in children with type 1 diabetes improved at a national level after years of noteworthy improvement in metabolic control? Is more optimal glycemic control associated with higher HRQOL? Is HRQOL better when diabetes technology is used?

The primary aim of this study was to analyse the association between diabetes-specific HRQOL and HbA1c in a cross-sectional study of children and adolescents with type 1 diabetes from the NCDR. The secondary aims were to evaluate associations of diabetes-specific HRQOL with age, sex, the use of insulin pumps, and CGM and to assess differences between self-reported and proxy-reported diabetes-specific HRQOL.

## 2. Methods

### 2.1. Study Population

In Norway, all children with diabetes up to 18 years of age receive diabetes care in a pediatric department. All pediatric departments report standardized data to the NCDR at diabetes onset and thereafter annually. The NCDR is a nationwide registry of prospective registration of newly diagnosed childhood-onset diabetes with a high ascertainment [12].

The cases in our study were classified as type 1 diabetes according to EURODIAB criteria [19]. The cohort represented a homogenous population, comprising mainly ethnic Norwegians [20]. Norway is a highly developed welfare state, both in terms of access to education and health care. Public schools provide education free of tuition fees, in order to give the same possibilities for education, regardless of economic and social background, age, gender, and physical disabilities. Pediatric diabetes care in Norway is solely given at hospitals with pediatric departments. Children and adolescents under 16 years of age have access to free medical health care, and adolescents 16 years or older pay only a limited contribution of approximately $200 per year for the sum of all required medical expenses, including all medical consultations and diabetes technology exceeding this amount. The onset of type 1 diabetes was defined as the date the subjects received insulin for the first time. Since 2002, patients in the NCDR have been screened for monogenic diabetes. Monogenic diabetes accounts for less than 2% of patients in this age group [21]. We excluded all individuals who were likely to have type 2 diabetes and those known to have monogenic diabetes. Details on the use of diabetes technology and incidence of acute complications such as severe hypoglycemia (2.6%) and diabetic ketoacidosis leading to hospitalization (3.5%) in this population have been published earlier [22]. Late diabetes complications are extremely rare in children and adolescents in Norway [12].

### 2.2. Measurements

For the evaluation of diabetes-specific HRQOL, we used the DISABKIDS condition specific module diabetes (DDM-10) questionnaire [23], which has two scales: the impact scale and the treatment scale. The impact scale (possible range score 0-100, based on six items) reflects emotional reactions of needing to control everyday life and to restrict one's diet. The treatment scale (possible range score 0-100, based on four items) refers to carrying equipment and planning treatment [24]. Each of the ten items assesses diabetes-specific quality of life using a 5-point Likert scale from 1=never to 5=always. Overall Cronbach's alpha values, described by the DISABKIDS group, were 0.84 for the impact scale and 0.85 for the treatment scale [24]. We tested the internal consistency for children and parents separately, resulting in an impact scale Cronbach's alpha value of 0.76 for all participating children and 0.78 for all participating parents. Cronbach's alpha for the treatment scale was 0.81 and 0.83, respectively. All items were negatively worded, and questions were therefore reverse-scaled before the impact and treatment scales were computed. Consequently, higher scores on the impact and treatment scales mean better health-related quality of life [24]. DDM-10 has been tested in a European reference group [24], and a Norwegian version of DISABKIDS has been tested for its reliability and validity [16]. Both the generic and the diabetes-specific modules have been used in several countries [17, 25, 26] and are routinely used in the Nordic countries [27].

All participants provided written informed consent to participate in the survey. Children and parents were asked to answer the questionnaire independently. The parent version of the questionnaire was completed by the mother, father, or both together. The child's age at examination, sex, actual use of insulin pump and/or CGM, and diabetes duration were part of the standardized annual data collection for the NCDR.

Blood samples were routinely taken at the yearly follow-up and the HbA1c of all children was analysed at the same DCCT standardized laboratory (Aker Laboratory, Department of Medical Biochemistry, Oslo University Hospital, Oslo, Norway).

The completion of the questionnaire and the collection of clinical data and blood samples were done at the same consultation.

### 2.3. Sampling Process

**Figure 1:**
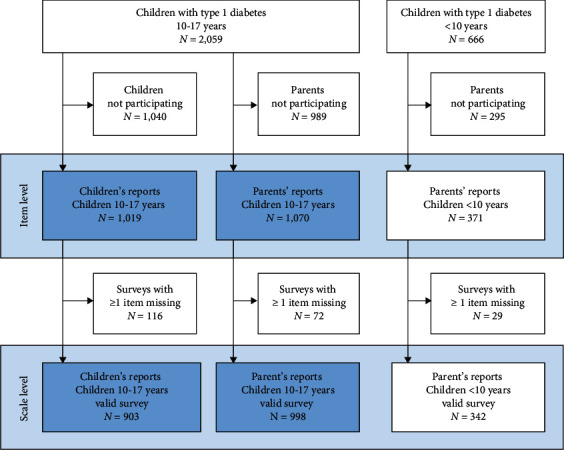
Flowchart of the sampling process. For statistics on item level, surveys from all participants were used (*N* = 1,019 + *N* = 1,070 + *N* = 371). For scales based on sum scores, we used only completed surveys (valid surveys, *N* = 903 + *N* = 998 + *N* = 342). When comparing children's and parents' responses, we only used responses from the same age group (marked in blue, 10-17 years).

The sampling process is presented in Figure 1. All children with type 1 diabetes aged 10 to 17 years (*N* = 2,059) were invited to participate in terms of completing self-reports about HRQOL by means of the DDM-10 questionnaire. Regardless of their child's age, all parents (*N* = 2,725) were invited to complete the same questionnaire in the corresponding proxy-report.

We received DDM-10 reports from 20 of 25 pediatric departments. Of 2,059 children aged 10-17 years, 1,019 (50%) participated. In total, 1,441 parents (53%) participated, there of 1,070 parents of children 10-17 years (52%) and 371 parents (56%) of under 10-year-old children. Of the participating 1,019 children aged 10-17 years, 903 answered the questionnaire completely. Of the 1,441 participating parents (998 parents of children aged 10-17 years and 342 parents of children under 10 years), 1,340 answered the questionnaire completely. These fully completed questionnaires were defined as the “valid survey”. The inferential analyses were based on participation and not on completed questionnaires (valid surveys, see Figure 1). Missing data on other variables were handled by listwise deletion.

### 2.4. Statistical Analysis

Differences in demographic characteristics, clinical profile, and treatment regimen of the registered children, and the participating and not participating children and parents were assessed by two-sample *t*-test (HbA1c, age, and diabetes duration) and chi-square test (sex, insulin pump, and CGM use). Mean group differences in the results from the DDM-10 questionnaire between participating children and parents were tested with two-sample *t*-tests.

For the analysis of the two scales, we only included completed questionnaires containing all items (“valid surveys”).

The association of the two DDM-10 scales (impact and treatment) with the child's HbA1c, age, sex, use of an insulin pump (vs. pen), and CGM (vs. self-measurement of blood glucose) were tested in a two-step regression model. First, we estimated the univariate model for each predictor variable, then we estimated the fully adjusted model including all predictor variables. Analyses were adjusted for the effects of variables that could confound or moderate the associations studied. Significance level was set to 0.05. All computation was done in R 4.0.4 [28].

The study was approved by the Regional Committee for Medical and Health Research Ethics (reg. no. 2016/1613/REC West) and registered in clinicaltrials.gov (ref. no. NCT04201171).

## 3. Results

### 3.1. Comparison between Participants and Nonparticipants

Table 1 shows the demographic and clinical information of the participating and not participating children and parents. The mean age of all children with type 1 diabetes registered in 2017 in the NCDR (*N* = 2,725) was 12.6 years (range 0.4-17.9 years), the mean diabetes duration was 5.1 years (SD 3.7) and 46% were females. There were no significant differences between the participants and nonparticipants of the survey regarding the children's HbA1c and the proportion of insulin pump or CGM usage (Table 1). Sex, age, and diabetes duration were different in the age group 10-17 years compared to the age group under 10 years. The age group 10-17 years was characterised by fewer females, younger age, and shorter diabetes duration among the participating children and parents than in the nonparticipating children and parents. In the age group under 10 years, participants and nonparticipants did not differ with regard to age, diabetes duration, and sex.

### 3.2. Health-Related Quality of Life

**Figure 2:**
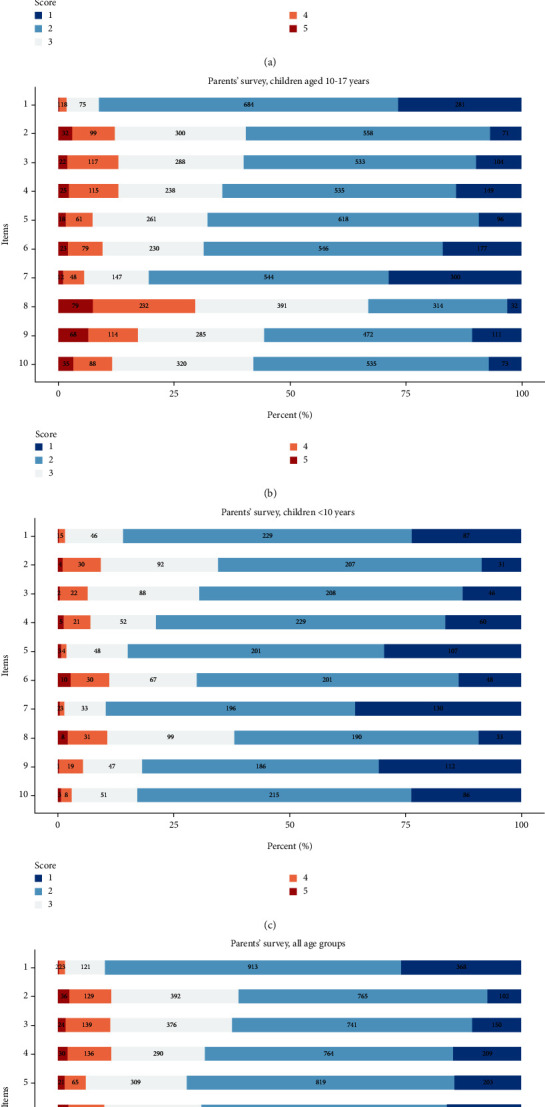
Stacked box plot with responses to items 1–10 for (a) children aged 10–17 years (*N* = 1,019), (b) parents of children aged 10–17 years (*N* = 1,070), (c) parents of children under 10 years (*N* = 371), and (d) all parents (*N* = 1,441). Valid *N* is marked in the bars. Blue reflects a higher quality of life.

Table 2 and Figure 2 show the survey results in detail. HRQOL reported by children and parents was generally high and highest in the parents' report of children under 10 years. In the valid analysis file, the mean score on the DISABKIDS impact scale was 69.6 in children aged 10-17 years (*N* = 903), 67.6 in parents of children aged 10-17 years (*N* = 998), and 71.3 in parents of children under 10 years of age (*N* = 342). The mean score of the DISABKIDS treatment scale was 65.2 in children aged 10-17 years, 62.1 in parents of children aged 10-17 years, and 74.2 in parents of children under 10 years of age. Parents of children aged 10-17 years gave lower scores on the impact scale (mean group difference = −1.8, p = 0.016) and treatment scale (mean group difference = −3.0, p = 0.002) than the participating children (Table 2).

On the item level, the score given by the children compared to the score given by the parents of the same age group (10-17 years) was statistically significantly lower on six of the ten items (Table 2), i.e., children reported higher levels of HRQOL than parents of the same age group of children did. Item 8 (measuring blood sugar) and item 9 (carrying the test equipment) showed the highest scores for both children and parents, indicating the lowest of HRQOL (Figure 2).

### 3.3. HbA1c

The mean HbA1c of all children with type 1 diabetes registered in the NCDR in 2017 was 7.8% (61.8 mmol/mol). The age group under 10 years had an approximately 0.5 percent point (pp) (i.e., 6 mmol/mol) lower mean HbA1c compared to the group of all registered children. In the age group of 10-17 year-olds, lower HbA1c was associated with higher HRQOL on both scales of the DDM-10 questionnaire, reported by both parents and children (*p* < 0.001).  Table 3 shows the results from unadjusted and adjusted models with HbA1c as predictor variable, and impact and treatment scales of the DDM-10 as outcome variables. In children 10-17 years, the impact score decreased with -0.26 (95% confidence interval (CI) for *B* = −0.35, -0.17) for every 1 mmol/mol of HbA1c increase, and the treatment score with -0.28 (95% CI for *B* = −0.40, -0.17) (*p* < 0.001). In parent's reports of children 10-17 years, impact score decreased with -0.27 (95% CI for *B* = −0.34, -0.19) for every 1 mmol/mol of HbA1c increase, and treatment score with -0.35 (95% CI for *B* = −0.45, -0.25), respectively (*p* < 0.001). Stratified data for CGM or pump use are shown in the Supplemental Figures 3–6.

### 3.4. Age

**Figure 3:**
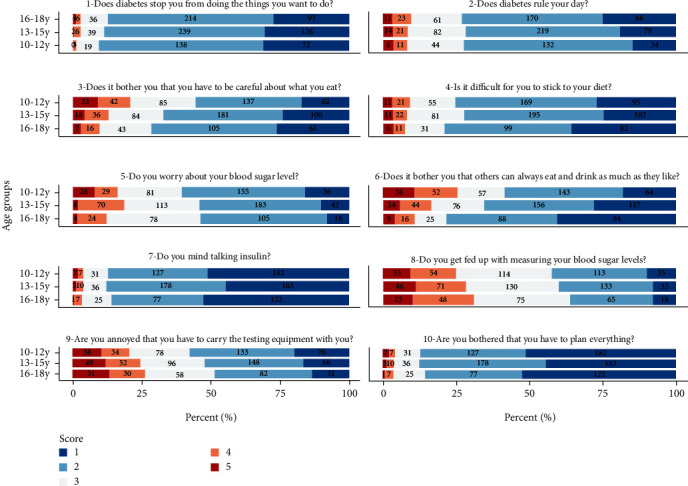
Stacked box plot with responses to items 1–10 for children aged 10–12, 13-15, and 16-18 years. Valid *N* is marked in the bars. Blue reflects a higher quality of life.

In the age group 10-17 years, older age was associated with a higher impact scale score in children's and parents' reports, but associated with a lower treatment scale score in children's reports. In the age group under 10 years, based only on the parents' reports, older age was associated with lower scores on both HRQOL scales. As shown in Table 3, multiple linear regression analysis of the age group 10-17 years revealed that children's higher age was associated with a higher impact score, both in children's reports (*B* = 0.82 (95% CI for *B* = 0.30, 1.35), *p* = 0.002) and parents' reports (*B* = 0.95 (95% CI for *B* = 0.52, 1.38), *p* < 0.001). Regarding the treatment scale, children's higher age was associated with a lower scale score in the children's reports (*B* = −0.69 (95% CI for *B* = −1.36, -0.02), *p* = 0.045). In the age group under 10 years, higher age was associated with a lower impact scale score (*B* = −1.57 (95% CI for *B* = −2.40, -0.74), *p* < 0.001) and the treatment scale score (*B* = −2.09 (95% CI for *B* = −3.03, -1.14), *p* < 0.001). Details regarding the response profile on item level in different age groups are presented in Table 3 and Figure 3.

### 3.5. Diabetes Duration

There was no association between diabetes duration and the impact or treatment scale score on any of the children's or parents' reports (Table 3).

### 3.6. Sex

Female sex was associated with lower HRQOL, reported as impact and treatment scale score by both children and parents in the age group 10-17 years. In the age group under 10 years, no associations between sex and HRQOL were found. Female sex in the age group 10-17 years was associated with lower impact scale score (*B* = −4.60 (95% CI for *B* = −6.84, -2.36), *p* < 0.001) and treatment (*B* = −8.02 (95% CI for *B* = −10.90, -5.13), *p* < 0.001) in the children's report. Parents' reports in the age group 10-17 years showed no significant association between female sex and impact scale score. The treatment scale score was associated with sex (*B* = −4.05 (95% CI for *B* = −6.42, -1.68), *p* < 0.001), but compared to the children's survey, the effect size was smaller in parents' reports in the age group 10-17 years (Table 3, Figure 2(b), and Supplemental figures 1 and 2).

### 3.7. Insulin Pump Use

Of all children registered in the NCDR in 2017 (*N* = 2,725), 74.2% were using an insulin pump. Pump usage was higher in the age group under 10 years (Table 1). Insulin pump usage was associated significantly with lower HRQOL on the treatment scale score in the parents' reports of the age group 10-17 years (*B* = −2.91 (95% CI for *B* = −5.62 − 0.20), *p* = 0.035), but not in any of the other groups (Table 3 and Supplemental figures 3 and 4). On the item level, insulin pump users reported in a significantly higher grade to “mind taking insulin” than pen users (item 7: 2.97 vs. 2.67, *p* < 0.001).

### 3.8. CGM Use

Of all children registered in the NCDR in 2017 (*N* = 2,725), 51.7% were using CGM. CGM usage was higher in the age group under 10 years (Table 1). There were no significant associations in any of the age groups between the use of CGM and the two scale scores of parents or children (Table 3 and Supplemental figures 5 and 6). CGM use was significantly associated with a lower item 8 score of the children's report, which relates to measuring blood sugar (*B* = −0.17 (95% CI for *B* = −0.31, -0.03) *p* = 0.018).

## 4. Discussion

HRQOL in our national cross-sectional cohort of children and adolescents with type 1 diabetes was generally high. There was a significant association between low HbA1c and HRQOL in the age group 10-17 years, but the effect size for this association was rather small. A clinically meaningful change in mean HbA1c of 0.5 pp (i.e., 6 mmol/mol) corresponds to a change of between 1.4 and 2.2 on mean DDM-10 scale scores (possible range: 0-100) in this age group. Surprisingly, HbA1c was not associated with parent-reported HRQOL in the age group under 10 years. Parents of children aged 10-17 years reported lower HRQOL for their children than the children and adolescents themselves. Our results regarding the child's age and HRQOL were heterogeneous in the different age groups, and across HRQOL scales and respondent groups. Male sex was clearly associated with better HRQOL in the age group 10-17 years, but not in the younger group. Regarding insulin pumps, their use had a negative association with HRQOL in the age group 10-17 years in the parents' report, but not otherwise. Use of CGM use was not associated with lower HRQOL.

To our knowledge, this is the first population-based study on HRQOL in children and adolescents with type 1 diabetes since 2013 [17]. The TEENs study has assessed HRQOL based on international, cross-sectional data of youth with type 1 diabetes collected in 2012 [7].

Differences in self-reports and proxy reports regarding diabetes related quality of life have been addressed earlier [29, 30]. Our results are in line with these reports, who found higher HRQOL was reported by youth than by parents. Our data are also generally in line with existing published data on the association between HRQOL and HbA1c [1, 2, 7, 17, 31]. However, regarding the younger age group under 10 years, we could show that HbA1c was not associated with how parents assessed their child's experience of the different aspects of having diabetes and the resulting HRQOL. On the contrary, both children themselves and parents of the age group 10-17 years reported higher impact and treatment scale scores (i.e., higher HRQOL) with lower HbA1c values (i.e., better metabolic control).

These age-related differences were even more obvious when we assessed the response of children in different age groups on DDM-10 item level: some items were given an equal response in different age groups, some were given a more or a less positive response. Higher age in the age group 10-17 was associated with a higher impact scale score, indicating that emotional reactions of needing to control everyday life and to restrict one's diet are getting less with advancing age (Figure 3). Older children might have ‘adapted' to their diabetes or have a better understanding of it, and thus, better be able to cope with their condition. There are, however, differences between females and males, as Supplemental figure 1 and 2 show. Regarding the treatment scale, increasing age was associated with lower HRQOL in the children's reports. This could be explained by the fact that children with increasing age are increasingly taking over the primary responsibility of managing their own diabetes (i.e., managing their own diet, counting carbohydrates, calculating insulin doses, monitoring glucose levels, etc.), which earlier, has been their caregiver's responsibility. As such, the children may face a greater burden as they transition to taking more care of themselves. In the age group under 10 years however, parents reported worse HRQOL on both scales the older the child gets. Diabetes duration does not show the same association. A possible interpretation could be that parents experience that the child gets more aware of restrictions due to their diabetes with increasing age, when at the same time children are expected to become more independent and autonomic.

There are several reports on the lower quality of life reported by females with type 1 diabetes [7, 32–34]. In addition, these sex differences have been described in healthy children and adolescents without type 1 diabetes or other chronic diseases [35]. Possible explanations have been proposed, such as females being more worried about their diabetes [32] or being demanding more of themselves [33], and improved clinical interventions have been suggested. Unfortunately, these sex differences remain in children 10-17 years of our population-based study sample, despite increasing metabolic control and a higher proportion of usage of insulin pumps and glucose sensors over the last decade.

The different aspects of HRQOL in the insulin pump user group, evaluated in detail by both the child and its parents, showed some interesting results. Despite the use of insulin pumps in approximately three quarters of the participants, insulin pump usage was either not associated or negatively associated (parents' reports, age 10.17 years) with HRQOL on both scales. Especially the fact that pump users reported a higher grade to ‘mind taking insulin' (item 7) than pen users was surprising and has to our knowledge not yet been described. A small longitudinal study on the use of insulin pumps and HRQOL from 2006 [4] did not show any significant improvements 15 months after pump therapy start. Children who started pump therapy showed an improvement of HRQOL after six months, compared to a control group, waiting for pump therapy [8]. A systematic review and meta-analysis on the possible effect of insulin pumps on HRQOL in pediatric patients [6] concluded that recommendations for pump therapy could not be made based on existing studies, due to their poor methodology, small sample sizes and short follow-up.

Our results regarding the use of CGM use were similar to the use of insulin pumps: Usage was not associated with HRQOL. Existing knowledge on the association between CGM use and HRQOL in the pediatric and adult population is limited. Polonsky et al. [36] found in a longitudinal study, with 24 weeks follow-up, an improvement in diabetes-specific QOL measures. However, the relevance of their findings for a longer timeframe is unclear. The choice of diabetes technology will always be based on individual preferences and personal needs. From our data, which are not longitudinal, no conclusion is possible whether HRQOL in our population has improved on an individual or general basis due to diabetes technology. It might, however, be of interest for the clinician that the use of pumps and CGM is not associated with better HRQOL compared to nonusers.

Taking into account the various positive aspects of both insulin pumps and CGM, we would have expected a higher score especially on the treatment scale in users of these devices. The impact of both insulin pumps and CGM on HRQOL might be transient, which would explain the discrepancy of our data to shorter longitudinal studies.

When comparing our data from 2017 to data collected from the NCDR in 2010 [17], the mean HbA1c in 2017 was lower (7.8% = 62 mmol/mol vs. 8.5% = 69 mmol/mol) and the percentage of children with HbA1c values below 7.5% (58 mmol/mol) was higher (39% vs. 18%) in 2017. Insulin pump usage in 2017 was more common (74% vs. 56%), and CGM usage was not even registered in 2010, since it was rarely used.

Children and parents reported in 2010 [17] a higher impact score (74/70) than in our 2017 data (69.6/67.6). This difference may not be clinically relevant, however, the lower HbA1c and the more frequent use of both insulin pumps and CGM in 2017 was not accompanied with better HRQOL related to control in everyday life and diet restrictions. In contrast, treatment scale scores of children's self-report reflecting HRQOL influenced by carrying equipment and planning treatment were slightly lower in 2010 (63) compared to our data from 2017 (65.2). This small difference is probably without any clinical relevance. Interestingly, the highest impact and treatment scale scores could be seen in the parents' reports in the age group under 10 years, which also has the highest proportion of pump and CGM usage. However, our results were clearly different from the original 2006 DISABKIDS European reference population for diabetes [24], with higher child-reported impact (69.6 vs. 62.7) and treatment scale (65.2 vs. 58.9) scores. A study from Germany in 2009 [26] showed a lower child-reported impact (66.2 vs. 69.6) and treatment (56.4 vs. 65.2) scale score. Compared with Swedish data collected in 2004-2005 [25], we saw a higher self-reported impact scale score (69.6 vs. 63), whereas treatment scale scores in our cohort were lower (65.2 vs. 68). Differences between Norwegian children's and parents' reports were smaller, both for impact [2] and treatment (3.1) scale scores, compared to Swedish data (impact: 8; treatment: 10). Regarding results from different time points and populations, comparing HRQOL scores should generally be done with caution, as stated by Symonds et al. [37]. However, the large difference between German and Norwegian Treatment scores from 2009 and 2017 (56.4 vs. 65.2) can support the nation that advances in diabetes treatment, such as the accessibility of insulin analogues and diabetes technology lead to better treatment related quality of life.

Our study has limitations. Generally, proxy-reported HRQOL may not represent the respondents' subjective experience. As described in the SEARCH for Diabetes in Youth study [29], parents of the younger age group tend to give higher scores than their children, whereas parents of the older age group tend to give lower scores than their children. Although the validity of proxy reports might be questionable, the parents' view can still be an interesting and important part of an evaluation of the child's HRQOL. Regarding the use of the DDM-10 questionnaire, some of the items still refer to therapy with pen and blood glucose measurement and may not adequately reflect the situation with a pump and a CGM. Furthermore, the DDM-10 does not measure HRQOL as a whole, but only diabetes-specific aspects of HRQOL. We did not assess the psychological status of the individuals in our cohort. However, mental health distress, which could confound the associations described in this study, has approximately equal prevalence in Norway as in other Northern European countries. Another limitation is the lack of CGM data. At the time of data collection in 2017, CGM data were not yet collected by the NCDR. The survey was performed in 2017 and the prevalence of CGM and pump use has increased since then. The subgroup using such technical devices in 2017 may have had higher severity of diabetes and more frequent complications than the subgroup of diabetes patients using such devices today. However, we argue that the nil-finding with regard to the studied association between use of technical devices and HRQOL in our study is still valid, as associations studied in a subgroup with higher illness severity (i.e., in 2017) would be expected to be stronger than the expected associations in a less severely ill sample at present. That is, weaker associations between the use of technical devices and HRQOL is expected today in patient groups with less severity using technical devices today.

Our study has several strengths. The study is nationwide and population-based, covering 98% of all children and adolescents up to 18 years of age with type 1 diabetes in Norway. A high number of children and parents (50-56% in the analysed groups) participated. Consequently, the generalizability of the study's findings to a Northern European population is very high. The study provides insight into the associations studied in different age groups, and the perspective of both children and parents.

Current clinical guidelines recommend the assessment of HRQOL in children and adolescents with type 1 diabetes [38, 39]. As diabetes treatment modalities and the level of reached metabolic control in the pediatric population are under continuous development, investigating diabetes-related quality of life, and its association with sex, age, and treatment options should be a regular part of clinical trials and registry work. For clinicians caring for children and adolescents with type 1 diabetes, knowledge on the association between HRQOL and HbA1c, sex, and the use of insulin pumps and CGM, as well as the different HRQOL evaluation of children themselves and their parents, can be valuable in their efforts of improving the HRQOL of their patients.

## Figures and Tables

**Table 1:** Demographic characteristics, clinical profiles, and treatment regimens of all children with type 1 diabetes registered in the Norwegian Childhood Diabetes Registry's (NCDR) annual registration in 2017, stratified by participating and nonparticipating children and parents.

	All registered children (NCDR annual registration)		Children 10–17 years (*N* = 2,059)						Children <10 years (*N* = 666)		
			Children participating	Children not participating	*p*	Parents participating	Parents not participating	*p*	Parents participating	Parents not participating	*p*
Valid *N*		2,725	1,019	1,040		1,070	989		371	295	
Proportion			49.5%	50.5%		52.0%	48.0%		55.7%	44.3%	
HbA1c	mmol/mol	61.8	63.3	63.8	0.49	63.2	63.9	0.22	56.4	56.0	0.61
	%	7.8	7.9	8.0		7.9	8.0		7.3	7.3	
Age	Years	12.6	14.1	14.6	<0.05	14.0	14.7	<0.05	7.3	7.2	0.64
Diabetes duration	Years	5.1	5.6	6.0	<0.05	5.5	6.1	<0.05	3.0	3.1	0.30
Females	%	45.7	41.9	50.4	<0.05	41.8	51.0	<0.05	44.2	44.1	1.00
Insulin pump users	%	74.2	73.7	71.1	0.22	73.0	71.8	0.61	79.1	80.3	0.78
CGM users	%	51.7	45.7	44.9	0.74	45.8	44.7	0.68	71.7	70.0	0.68

Group differences between participants and nonparticipants were tested using two-sample *t*-tests (HbA1c, age, and diabetes duration) or chi-squared tests (gender, pump use, and CGM use).

**Table 2:** Results from the DISABKIDS DDM-10 survey: mean for and mean group differences between children and parents.

Item	Children 10–17 years						Children <10 years	
	Children's report		Parents' report		Group		Parents' report	
	*N* = 1,019		*N* = 1,070		Difference		*N* = 371	
	*N*	Mean (SD)	*N*	Mean (SD)	Mean	*p*	*N*	Mean (SD)
Impact scale	903	69.6 (16.8)	998	67.6 (14.5)	−1.8	0.016	342	71.3 (13.8)
(1) Does diabetes stop you from doing the things you want to do?	1,001	1.85 (0.69)	1,059	1.84 (0.62)	−0.00	0.868	368	1.92 (0.66)
(2) Does diabetes rule your day?	997	2.20 (0.93)	1,060	2.49 (0.87)	+0.30	<0.0001	364	2.37 (0.80)
(3) Does it bother you that you have to be careful about what you eat?	1,010	2.35 (1.10)	1,064	2.45 (0.89)	+0.11	0.015	366	2.25 (0.77)
(4) Is it difficult for you to stick to your diet?	991	2.08 (0.96)	1,062	2.37 (0.93)	+0.29	<0.0001	367	2.13 (0.80)
(5) Do you worry about your blood sugar level?	994	2.53 (0.99)	1,054	2.32 (0.79)	−0.20	<0.0001	363	1.88 (0.73)
(6) Does it bother you that others can always eat and drink as much as they like?	1,003	2.32 (1.19)	1,055	2.27 (0.90)	−0.05	0.251	356	2.31 (0.91)

Treatment scale	903	65.2 (21.6)	998	62.1 (18.8)	−3.0	0.002	342	74.2 (15.5)
(7) Do you mind taking insulin?	998	1.69 (0.84)	1,051	1.98 (0.84)	+0.29	<0.0001	364	1.77 (0.69)
(8) Do you get fed up with measuring your blood sugar levels?	990	2.89 (1.11)	1,048	3.01 (0.97)	+0.12	0.012	361	2.42 (0.86)
(9) Are you annoyed that you have to carry the testing equipment with you?	998	2.65 (1.23)	1,050	2.58 (1.03)	−0.07	0.146	365	1.93 (0.82)
(10) Are you bothered that you have to plan everything?	998	2.37 (1.13)	1,051	2.50 (0.87)	+0.13	0.004	363	1.97 (0.74)

Low item scores and high scale scores (impact and treatment) indicate high quality of life. Mean group differences between children and parents were tested using two-sample *t*-tests. Due to rounding, mean group differences might deviate slightly.

**Table 3:** Associations between Impact and Treatment scale scores and HbA1c, age, diabetes duration, sex, use of insulin pump and CGM in the valid sample (N =903 [children's reports, age 10-17], N =998 [parent's reports, age 10-17], N =342 [parent's reports, age<10years]). Effect sizes beta (B) with 95% confidence intervals. Unadjusted and fully adjusted multivariate linear regression models.

	Children 10-17 years				Children <10 years	
	Children's report		Parents' report		Parents' report	
Impact scale	B (95% CI), p		B (95% CI), p		B (95% CI), p	
	Unadjusted	Adjusted	Unadjusted	Adjusted	Unadjusted	Adjusted
HbA1c [mmol/Mol]	-0.24 (-0.32, -0.15), p <0.001	-0.26 (-0.35, -0.17), p <0.001	-0.23 (-0.30, -0.16), p <0.001	-0.27 (-0.34, -0.19), p <0.001	-0.13 (-0.32, 0.07), p =0.212	-0.08 (-0.29, 0.12), p =0.421
Age [years]	0.81 (0.32, 1.30), p =0.001	0.82 (0.30, 1.35), p =0.002	0.89 (0.49, 1.30), p <0.001	0.95 (0.52, 1.38), p <0.001	-1.54 (-2.27, -0.80), p <0.001	-1.57 (-2.40, -0.74), p <0.001
Duration [years]	0.06 (-0.24, 0.36), p =0.694	0.13 (-0.19, 0.45), p =0.423	-0.07 (-0.18, 0.32), p =0.591	0.14 (-0.13, 0.40), p =0.316	-0.48 (-1.22, 0.26), p =0.201	0.17 (-0.66, 1.00), p =0.692
Female sex	-5.04 (-7.25, -2.83), p <0.001	-4.60 (-6.84, -2.36), p <0.001	-2.60 (-4.42, -0.78), p =0.005	-1.56 (-3.39, 0.26), p =0.093	-1.34 (-4.31, 1.63), p =0.375	-1.51 (-4.49, 1.47), p =0.319
Use of insulin pump	-2.46 (-4.93, 0.02), p =0.052	-0.92 (-3.46, 1.62), p =0.479	-2.79 (-4.84, -0.75), p =0.007	-2.02 (-4.11, 0.06), p =0.057	1.72 (-1.92, 5.37), p =0.353	3.28 (-0.58, 7.14), p =0.096
Use of CGM	-2.73 (-4.96, -0.50), p =0.016	-2.23 (-4.50, 0.03), p =0.053	-2.16 (-3.99, -0.32), p =0.021	-1.63 (-3.47, 0.22), p =0.084	-2.08 (-5.39, 1.23), p =0.218	-3.32 (-6.76, 0.11), p =0.058

Treatment scale	B (95% CI), p		B (95% CI), p		B (95% CI), p	
	Unadjusted	Adjusted	Unadjusted	Adjusted	Unadjusted	Adjusted
HbA1c [mmol/Mol]	-0.32 (-0.43, -0.21), p <0.001	-0.28 (-0.40, -0.17), p <0.001	-0.36 (-0.45, -0.27), p <0.001	-0.35 (-0.45, -0.25), p <0.001	-0.06 (-0.28, 0.17), p =0.617	-0.01 (-0.22, 0.24), p =0.955
Age [years]	-0.76 (-1.40, -0.13), p =0.018	-0.69 (-1.36, -0.02), p =0.045	-0.45 (-0.97, 0.08), p =0.098	-0.35 (-0.90, 0.20), p =0.216	-1.86 (-2.69, -1.04), p <0.001	-2.09 (-3.03, -1.14), p <0.001
Duration [years]	-0.34 (-0.73, 0.05), p =0.088	-0.06(-0.47, 0.35), p =0.781	-0.12 (-0.44, 0.21), p =0.476	0.21 (-0.14, 0.55), p =0.236	-0.35 (-1.18, 0.48), p =0.404	0.38 (-0.56, 1.32), p =0.423
Female sex	-7.53 (-10.36, -4.69), p <0.001	-8.02 (-10.90, -5.13), p <0.001	-4.36 (-6.71, -2.01), p <0.001	-4.05 (-6.42, -1.68), p <0.001	0.07 (-3.28, 3.42), p =0.967	-0.09 (-3.46, 3.29), p =0.960
Use of insulin pump	-3.48 (-6.68, -0.28), p =0.033	-2.36 (-5.63, 0.92), p =0.158	-3.60 (-6.24, -0.96), p =0.008	-2.91(-5.62, -0.20), p =0.035	2.15 (-1.96, 6.27), p =0.304	1.65 (-2.74, 6.03), p =0.460
Use of CGM	1.91 (-0.97, 4.80), p =0.194	1.48 (-1.44, 4.39), p =0.320	-0.10 (-2.48, 2.28), p =0.933	-0.66 (-3.06, 1.73), p =0.587	-0.87 (-4.59, 2.84), p =0.645	-1.74 (-5.63, 2.16), p =0.381

## Data Availability

The registry data used to support the findings of this study have not been made available due to ethical rules of the Norwegian Childhood Diabetes Registry and Patient Privacy.

## Supplementary Materials

Supplementary Materials Figure 1: impact and treatment scale scores vs. age, stratified for sex. Figure 2: impact and treatment scale scores vs. HbA1c, stratified for sex. Figure 3: impact and treatment scale scores vs. scores vs. age, stratified for pump/pen use. Figure 4: impact and treatment scale scores vs. HbA1c, stratified for pump/pen use. Figure 5: impact and treatment scale scores vs. age, stratified for CGM use. Figure 6: impact and treatment scale scores vs. HbA1c, stratified for CGM use.
