# Supplementary material for: Relation of Health-Related Quality of Life with Glycemic Control and Use of Diabetes Technology in Children and Adolescents with Type 1 Diabetes: Results from a National Population Based Study
Source: J Diabetes Res. 2022 Nov 3;2022:8401328. doi: 10.1155/2022/8401328 (PMC9649325; doi:10.1155/2022/8401328)

**Supplemental figures**

Figure 1: Impact and Treatment scale scores vs age, stratified for sex


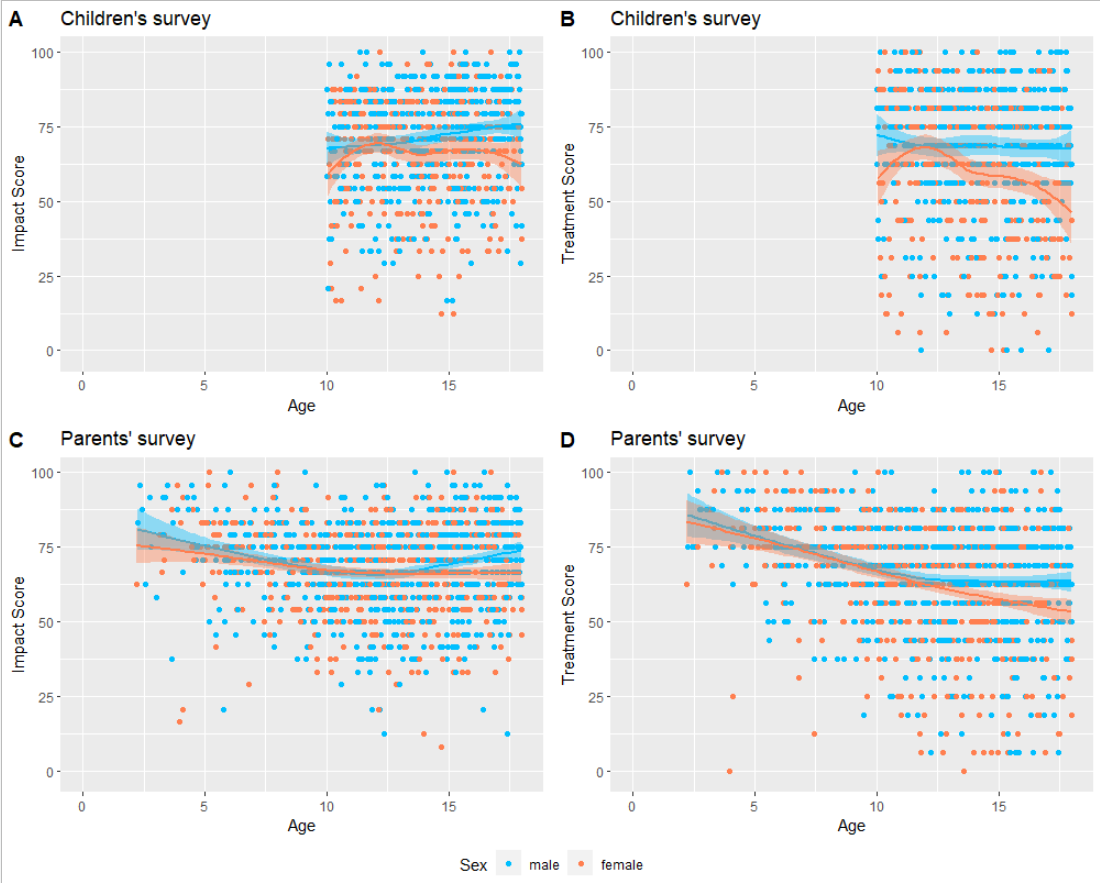


Figure 2: Impact and Treatment scale scores vs HbA1c, stratified for sex


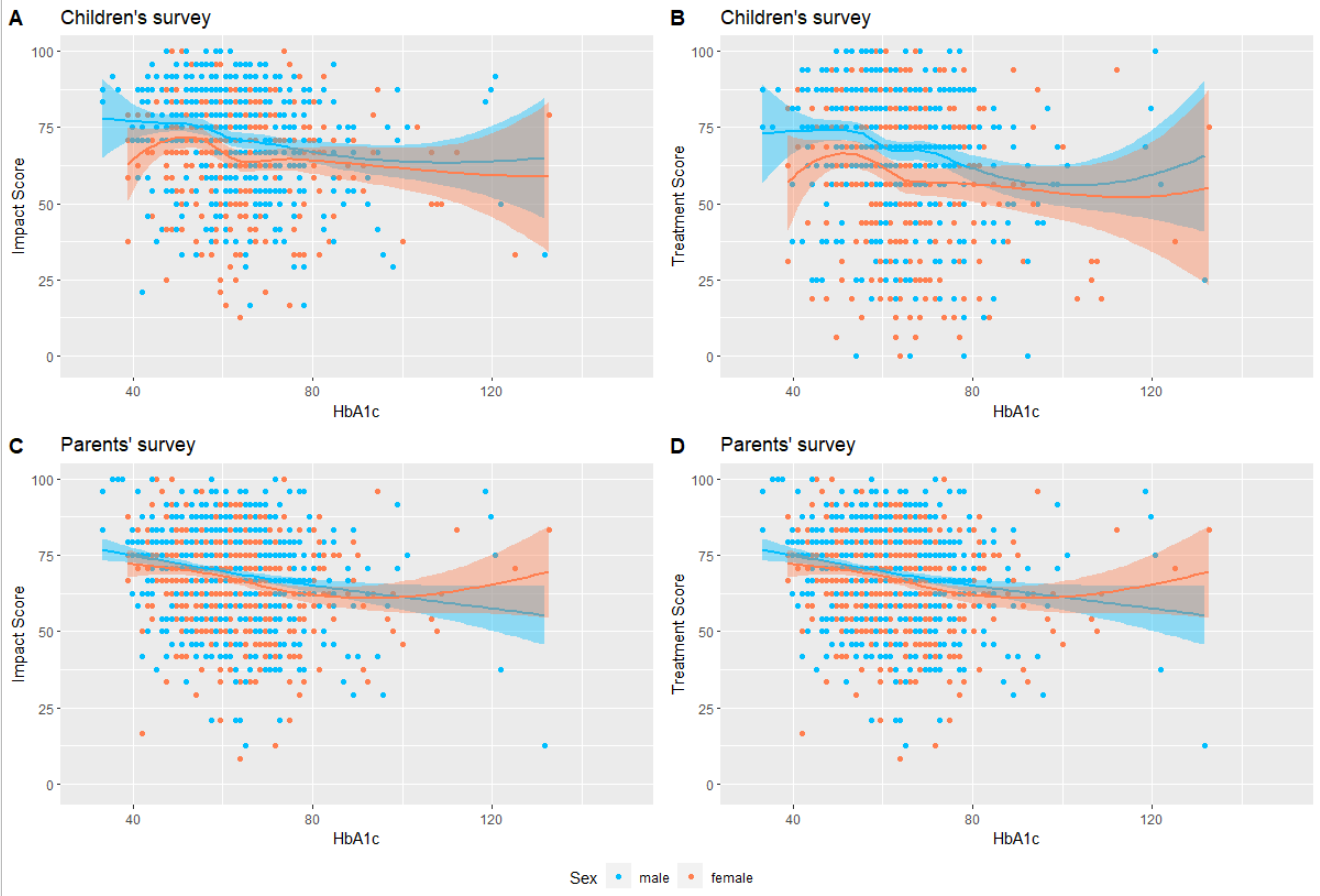


Figure 3: Impact and Treatment scale scores vs age, stratified for pump/pen use


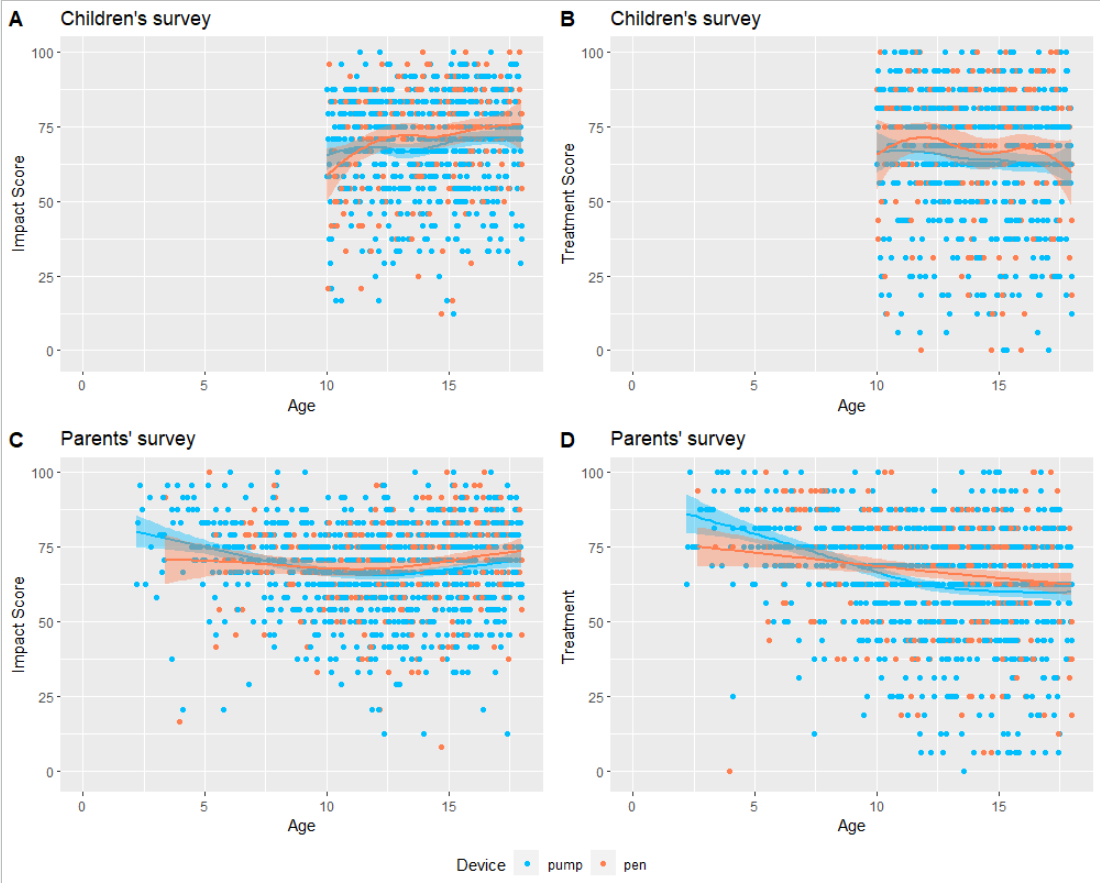


Figure 4: Impact and Treatment scale scores vs HbA1c, stratified for pump/pen use
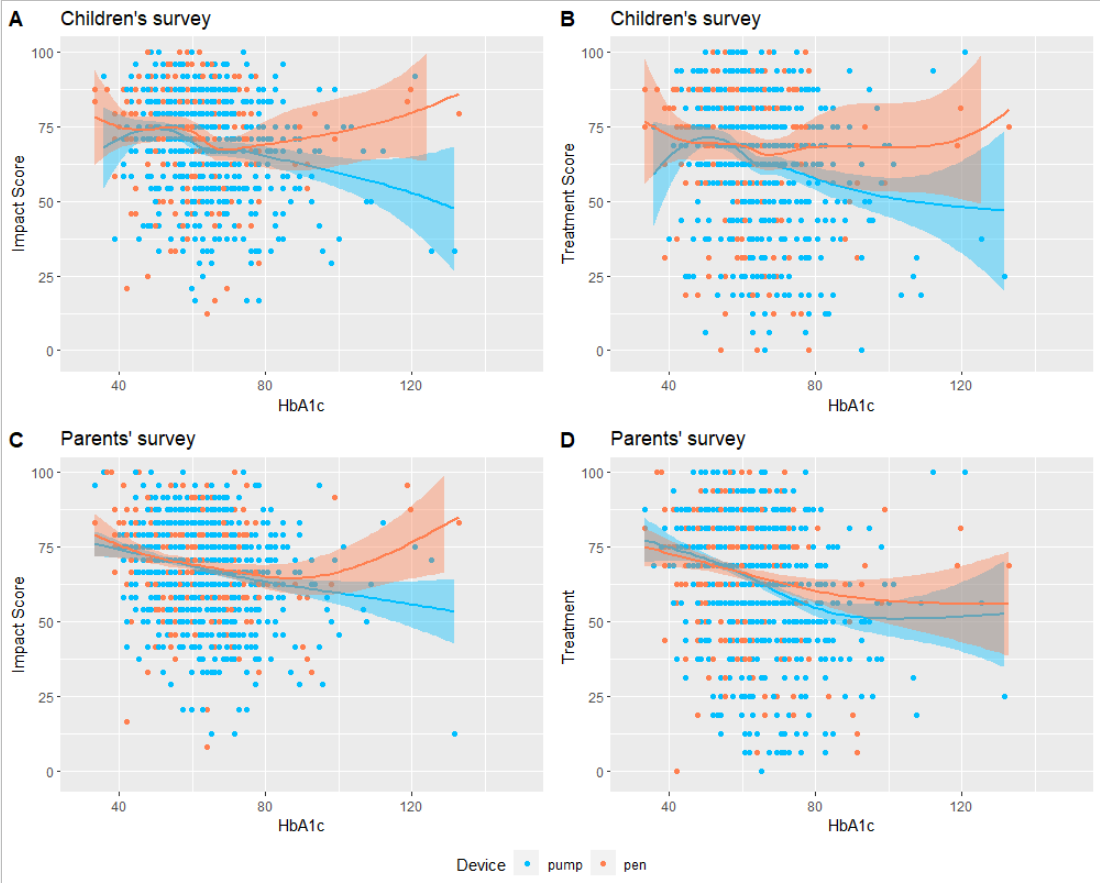


Figure 5: Impact and Treatment scale scores vs age, stratified for CGM use
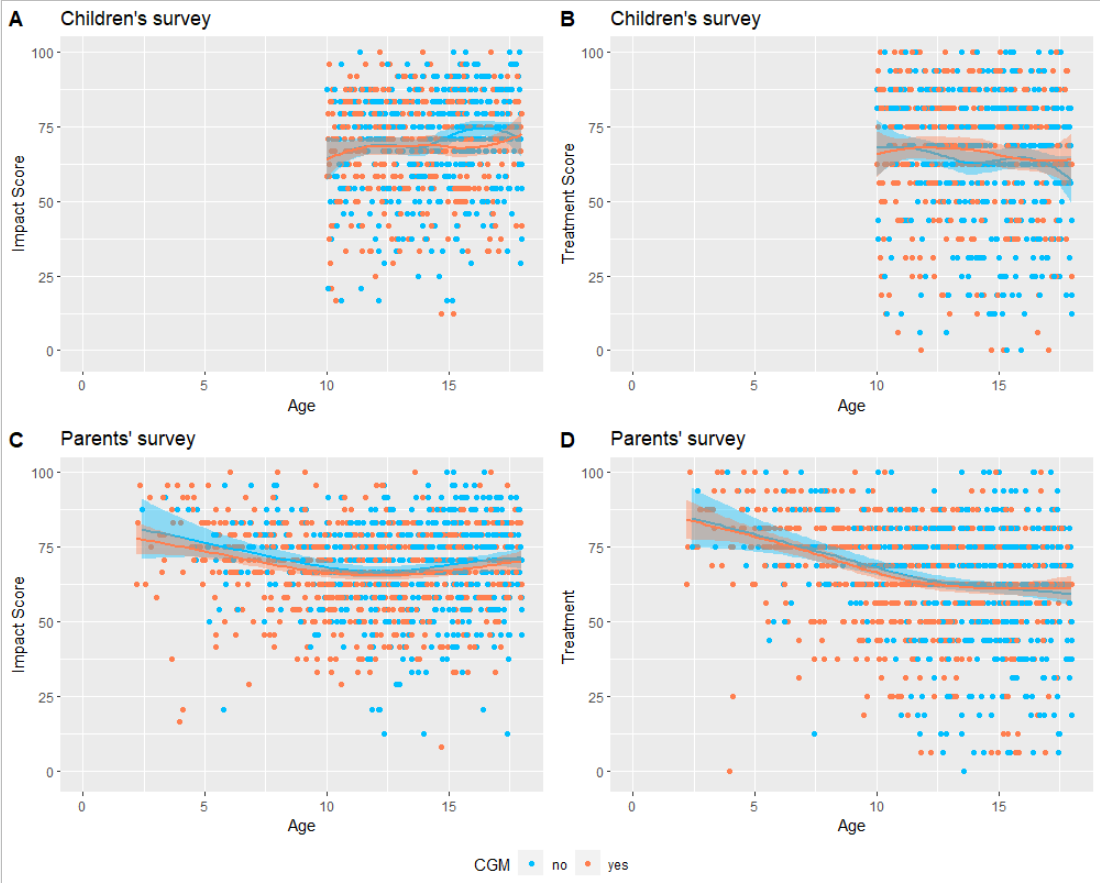


Figure 6: Impact and Treatment scale scores vs HbA1c, stratified for CGM use
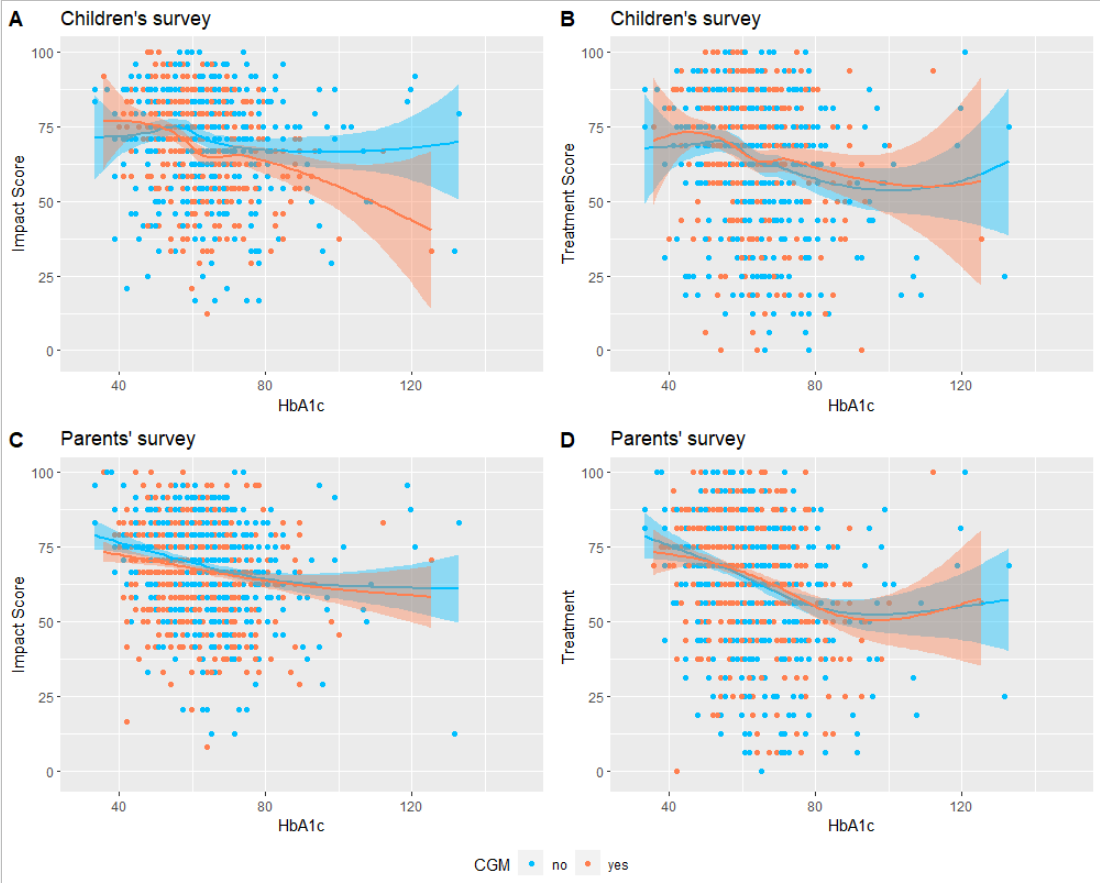

Supplement: Supplementary Materials — Figure 1: impact and treatment scale scores vs. age, stratified for sex. Figure 2: impact and treatment scale scores vs. HbA1c, stratified for sex. Figure 3: impact and treatment scale scores vs. scores vs. age, stratified for pump/pen use. Figure 4: impact and treatment scale scores vs. HbA1c, stratified for pump/pen use. Figure 5: impact and treatment scale scores vs. age, stratified for CGM use. Figure 6: impact and treatment scale scores vs. HbA1c, stratified for CGM use. [file 8401328.f1.docx]
